# PPARγ-dependent and -independent regulation of genes involved in hepatic methionine cycle in fasted or diet-induced obese mice

**DOI:** 10.1371/journal.pone.0358698

**Published:** 2026-09-18

**Authors:** Izabela Hawro, Samuel M. Lee, Rhonda D. Kineman, Jose Cordoba-Chacon

**Affiliations:** 1 Department of Medicine, Division of Endocrinology, Diabetes and Metabolism, University of Illinois Chicago, Chicago, Illinois, United States of America; 2 Research and Development Division, Jesse Brown VA Medical Center, Chicago, Illinois, United States of America; Zhejiang University of Technology, CHINA

## Abstract

Metabolic dysfunction-associated steatohepatitis (MASH) is associated with increased expression of hepatocyte peroxisome proliferator-activated receptor gamma (PPARγ, *Pparg*) and reduced expression of hepatic genes involved in the methionine cycle. The nuclear receptor PPARγ is activated by fatty acids, and we have shown that the knockout of *Pparg* in hepatocytes (*Pparg*^ΔHep^) reduces the negative effects of MASH on the metabolism of methionine. Here, we sought to determine whether hepatocyte *Pparg* is required for the transcriptional regulation of genes involved in the methionine cycle in conditions with altered fatty acid flux to the liver: fasting, refeeding, and high-fat diet (HFD)-induced obesity/steatosis. Fasting increased the expression of key genes involved in the methionine cycle, whereas 6h-refeeding reversed these effects and reduced the expression of phosphatidylethanolamine N-methyltransferase (*Pemt)* and cystathionine beta synthase (*Cbs)*. Although fasting increased hepatocyte *Pparg* expression, *Pparg*^ΔHep^ did not enhance the fasting and refeeding-mediated regulation of methionine cycle gene expression. We previously reported that diet-induced steatosis increased hepatocyte *Pparg* expression, and here we show that PPARγ-specific agonist rosiglitazone (RSG) reduced the expression of betaine homocysteine S-methyltransferase (*Bhmt)* and *Cbs* in diet-induced obese control mice. The PPARγ-dependent reduction of hepatic *Bhmt* and *Cbs* expression was confirmed in mouse primary hepatocytes. Interestingly, *Pparg*^ΔHep^ increased the expression of *Pemt* in HFD-fed mice and that of key genes of the methionine cycle in RSG-treated obese mice, including *Pemt, Bhmt* and *Cbs*, suggesting that *Pparg* negatively regulates their expression in the liver*.*

## Introduction

Methionine is an essential sulfur-containing amino acid that is used for protein synthesis or by the methionine adenosyltransferases 1a and 2a (*Mat1a* and *Mat2a*) to generate S-adenosylmethionine (SAM). The SAM-dependent methyltransferases methylate a wide range of substrates, including DNA, lipids, and amino acids, to maintain cellular health. For instance, glycine N-methyltransferase (*Gnmt*) uses SAM to convert glycine into sarcosine, and phosphatidylethanolamine N-methyltransferase (*Pemt*) needs three molecules of SAM to synthesize phosphatidylcholine from phosphatidylethanolamine. The transmethylation reactions of SAM produce S-adenosylhomocysteine (SAH), which is then converted into homocysteine by the adenosylhomocysteinease (*Ahcy*). Finally, homocysteine is processed by the cystathionine β-synthase (*Cbs*) in the transsulfuration pathway to produce glutathione, or remethylated by the 5-methyltetrahydrofolate-homocysteine methyltransferase (*Mtr*) or the betaine homocysteine methyltransferase (*Bhmt*) to regenerate methionine [1,2]. Of note, the expression of *Mat1a, Gnmt, Pemt, Ahcy, Bhmt,* and *Cbs* is high and almost exclusive to hepatocytes, and most of the whole-body methionine adenosyltransferase activity is found in the liver [1,3–5]. In fact, up to 85% of SAM-dependent methylation reactions and nearly 50% of methionine metabolism take place in the liver [6].

Diet-induced obesity and the progression of metabolic dysfunction-associated steatotic liver disease (MASLD) reduce the expression of *Mat1a*, *Gnmt, Pemt*, nicotinamide N-methyltransferase (*Nnmt*), *Ahcy*, and *Bhmt* in the liver [7–9]. Notably, the knockout of *Gnmt*, *Pemt*, or *Bhmt* leads to steatosis and progression of MASLD to metabolic dysfunction-associated steatohepatitis (MASH) [10–12], suggesting a critical role for methionine metabolism in maintaining liver health. Moreover, MASLD and MASH are associated with increased hepatic expression of the nuclear receptor peroxisome proliferator-activated receptor gamma (*Pparg*) in mice and humans [13–16]. Interestingly, early studies show that expression and pharmacological activation of *Pparg* in hepatocytes contribute to the development of steatosis in mouse models [14,17–22]. Furthermore, we have reported that expression of *Pparg* in hepatocytes contributes to the development of diet-induced MASH [23]. Specifically, adult-onset, hepatocyte-specific *Pparg* knockout (*Pparg*^ΔHep^) reduced the progression of diet-induced liver injury (hepatocyte ballooning, inflammation, and fibrosis). Some of the protective effects of *Pparg*^ΔHep^ might be due to preservation of the methionine cycle, where expression of hepatic methyltransferases (*Pemt or Bhmt*) is reduced by diet-induced MASH and restored by *Pparg*^ΔHep^ [16,24]. Notably, the transcriptional activity of PPAR in the liver is enhanced by fatty acids and also by specific pharmaceutical drugs called thiazolidinediones [25,26]. In our previous studies, we used rosiglitazone (RSG) to activate hepatocyte PPARγ because it is a potent and selective PPARγ agonist. However, it remains to be determined whether the activation of PPARγ in the liver reduces the expression of genes involved in the methionine cycle and, if so, whether that might contribute to the progression of MASLD to MASH.

In this study, we sought to explore if the expression of *Pparg* in hepatocytes contributes to the transcriptional regulation of genes involved in the methionine cycle under different metabolic states in which hepatic methionine metabolism is regulated, and fatty acid flux to the liver is altered. Of note, fasting increases the expression of hepatic genes involved in the methionine cycle [27–29], and diet-induced MASH may have a negative impact on the regulation of the hepatic methionine cycle [8]. Thus, we used liver samples from mice subjected to 24h fasting, with or without 6h of refeeding. Furthermore, we used liver samples from mice with diet-induced insulin resistance, obesity, and steatosis but without MASH [7]. Under these metabolic conditions, we have assessed the effect of hepatocyte *Pparg* expression and that of RSG-mediated activation of hepatocyte PPARγ on the transcriptional regulation of genes involved in the hepatic methionine cycle.

## Materials and methods

### Mouse studies

Animal studies were conducted with the approval of the University of Illinois Chicago (UIC) and Jesse Brown VA Medical Center (JBVAMC) Institutional Animal Care and Use Committee (IACUC). For these studies, PPARγ floxed mice in a C57BL/6J background were maintained as a homozygous breeding colony (*Pparg*^fl/fl^, Strain B6.129-Pparg^tm2Rev^/J, stock number 004584, Jackson Laboratories, Bar Harbor, ME). In the following studies, mice were monitored weekly throughout long-term experiments on special diets. We monitored the overall body condition and ability to ambulate of the mice in these studies. In addition, body weight was recorded every 4 weeks and if a mouse lost more than 20% of the average body weight of its diet-matched littermates (within the same group), it would have been euthanized by CO_2_ asphyxiation followed by cervical dislocation. No mouse used in this study met these humane endpoints. *Pparg*^ΔHep^ mice were generated by injection of adeno-associated virus serotype 8 (AAV8) with a thyroxine-binding globulin (TBG)-promoter driving Cre recombinase (AAV8-TBGp-Cre, Penn Vector Core, University of Pennsylvania, and Addgene, Watertown, MA, USA) into the lateral tail vein of *Pparg*^fl/fl^ mice as we previously reported [7,13]. An injection of AAV8-TBGp-Null (Penn Vector Core and Addgene) in *Pparg*^fl/fl^ mice was used to generate control mice.

#### Fasting and refeeding.

*Pparg*^fl/fl^ mice were housed in a temperature (22–24°C) and humidity-controlled specific pathogen-free barrier facility (JBVAMC animal facility) with 12h light/ 12h dark (lights on 0600h) and fed a standard chow diet (Formulab Diet 5008, Purina Mills, Richmond, IN) *ad libitum*. At 10–12 weeks of age, *Pparg*^fl/fl^ mice were injected with 1.5*10^11^ genome copies of AAV8-TBGp-Cre or AAV8-TBGp-Null [13]. One week after AAV injection, chow-fed control and *Pparg*^ΔHep^ mice were fasted for 24 hours before euthanasia by decapitation without anesthesia (food removed at 1200h). A subset of 24h-fasted mice from each group (food removed at 0700h), were then refed with a chow diet at 0700h, and euthanized by decapitation without anesthesia 6h later at 1300h. No mice reached the humane endpoints described in our IACUC protocol, and all the mice used in this study completed the experiment. To determine if fasting and refeeding altered hepatic gene expression from or to a “fed-like” state, liver samples from a previously published study [13] were used in which fed mice were euthanized by decapitation without anesthesia in the post-absorptive state, 4h after food withdrawal at 0700h. Blood glucose levels were determined using Blood Glucose Meter and test strips (Accu-Check ®, Roche), and trunk blood was collected in EDTA-coated tubes to obtain plasma and stored at −20°C. Tissues were weighed and rapidly snap-frozen in liquid nitrogen for molecular analyses. All the liver samples used for this study were obtained from littermate mice, which were simultaneously euthanized from 1100h to 1300h.

#### Diet-induced obese mice.

To examine the impact of diet-induced insulin resistance, obesity, and steatosis on the expression of hepatic genes involved in the metabolism of methionine in the presence and absence of hepatocyte *Pparg*, liver samples from a previously published study were used [7]. In brief, *Pparg*^fl/fl^ mice were housed in a temperature (22–24°C) and humidity-controlled specific pathogen-free barrier facility with 14 h light/ 10 h dark (UIC animal facility). Four- to six-week-old male *Pparg*^fl/fl^ littermate mice were switched from a standard chow diet to either a low-fat diet (LF, LFD) containing 10% kcal from fat (D12450J, Research Diets, New Brunswick, NJ, USA) or a high-fat diet (HFD) containing 60% kcal from fat (D12492, Research Diets) for 16 weeks. These diets are nutrient-matched and contain the same amount and composition of amino acids. Then, AAV vectors were intravenously injected to generate control and *Pparg*^ΔHep^ mice. One week later, a subset of HFD-fed control and *Pparg*^ΔHep^ mice were switched to an HFD supplemented with 70 mg of RSG maleate/kg of diet (HFD/RSG, Cat# D18061406, Research Diets) for 6 additional weeks. Three out of fifty four mice died unexpectedly without displaying the criteria for human endpoint indicated above. All the groups were euthanized by decapitation without anesthesia 4h after food withdrawal at 0700h. Livers were weighed and snap-frozen in liquid nitrogen for molecular analyses.

### Mouse primary hepatocytes (MPH)

MPH were obtained from 4 different groups: 1) **CHOW**- chow-fed 13- to 20-week-old male *Pparg*^fl/fl^ mice, 2) **LFD** – male *Pparg*^fl/fl^ mice injected with AAV8-TBGp-Null at 11−14 weeks of age and fed a LFD (D12450J) for 25−26 weeks, 3) **HFC + Fr** – male *Pparg*^fl/fl^ mice that were treated with AAV8-TBGp-Null at 12−13 weeks of age and fed a HFD containing 60% kcal from fat and 2% cholesterol (HFC, D12120101, Research Diets) supplemented with 10% fructose in the drinking water (HFC + Fr) for 24 weeks, 4) **HFC + Fr KO** – male *Pparg*^fl/fl^ mice that were injected with AAV8-TBGp-Cre at 11−13 weeks of age to generate *Pparg*^ΔHep^ and fed a HFC + Fr diet for 25−26 weeks. Mice from these four groups were anesthetized with ketamine/xylazine (100/10 mg/kg) and euthanized by exsanguination during the perfusion of the liver. MPH were isolated as previously described [24] with slight modifications. The liver was perfused using a perfusion system for cell isolation from liver (Harvard Apparatus, Holliston, MA) to control flow rate, and temperature at 37°C. First, we perfused up to 30 ml of Hanks’ Balanced Salt Solution without calcium or magnesium supplemented with EDTA (137 mM NaCl, 5.33 mM KCl, 0.44 mM KH_2_PO_4_, 0.33 mM Na_2_HPO_4_, 0.5 mM EDTA, pH 7.3) at 3.12 ml/min. Then, 12.5 to 20 ml DMEM without sodium pyruvate but supplemented with HEPES and collagenase (4.5 g/l glucose, 110 mM NaCl, 5.33 mM KCl, 44 mM NaHCO_3_, 0.7 mM Na_2_HPO_4_·H2O, 97.67mM MgSO_4_, 1.8 mM CaCl_2_, 15 mM HEPES, 25 μg/ml collagenase type I and II Liberase TM, (Sigma Aldrich, St. Louis, MO), pH 7.3) was perfused at 3.12 ml/min. When digestion was completed, livers were gently dissociated using cell scrapers in ice-cold DMEM/F-12 supplemented with 10% fetal bovine serum, 2 mM L-glutamine, 1x penicillin-streptomycin (complete DMEM/F-12). The cell suspension was filtered gently through 70 µm nylon strainers and centrifuged (5 min, 100g, 4°C) to pellet the hepatocytes, that were washed three times and centrifuged (5 min, 100g, 4°C) with fresh ice-cold complete DMEM/F-12. If cell viability determined by trypan blue exclusion was above 70%, viable hepatocytes were purified by density separation with 45% Percoll (Cytiva, Marlborough, MA) in phosphate-buffered saline buffer by centrifugation (20 min, 100g, 4°C, without deceleration). Isolated hepatocytes were resuspended in ice-cold complete DMEM/F-12 and plated at 2*10^5^ cells/well on 12-well plates precoated with type I rat tail collagen (Corning, Corning, NY). After 4 hours at 37°C in a humidified incubator with 5% CO_2_, the medium was replaced with fresh complete DMEM/F12 with and without 1 μM RSG (Sigma-Aldrich), and the cells were incubated for an additional 24 hours at 37°C with 5% CO_2_.

### Hepatic lipid extraction, hepatic and plasma metabolic endpoint assays

Hepatic lipids were extracted using isopropyl alcohol as we previously described [30]. Plasma non-esterified fatty acids (NEFA) and plasma and hepatic triglycerides (TG) were measured using colorimetric assays (Fujifilm Wako Diagnostics, Richmond, VA). Plasma insulin levels were determined using a commercial ELISA kit (Mercodia, Uppsala, Sweden). All assays were performed according to the manufacturer’s protocols.

### RNA isolation, cDNA synthesis, and real-time quantitative PCR (qPCR)

Hepatic and MPH RNA was extracted using Invitrogen™ TRIzol™ Reagent (Invitrogen, Carlsbad, CA) according to the manufacturer’s protocol. Extracted total RNA was treated with RQ1 RNase-Free DNase (Promega, Madison, WI). DNA-free RNA was then reverse-transcribed into cDNA using the First Strand cDNA Synthesis Kit (Thermo Scientific, Waltham, MA). A Real-Time qPCR reaction was performed using the Brilliant III Ultra-Fast QPCR Master Mix (Agilent Technologies, Santa Clara, CA). Specific primer sequences (Table 1) were used to amplify target genes. Details about the qPCR procedure, standard curves generation, and calculation were previously described [30,31]. mRNA copy number was adjusted by a normalization factor calculated from the mRNA copy number of at least two separate housekeeping genes (β-actin [*Actb*], and cyclophilin-A [*Ppia*] mRNA) using GeNorm 3.3 software [30,32].

**Table 1:** Sequence of qPCR primers used in this study.

Gene Name	NCBI Ref Seq acc#	Forward sequence	Reverse Sequence	Product size (bp)
*Actb*	NM_007393.3	CTGGGACGACATGGAGAAGA	ACCAGAGGCATACAGGGACA	205
*Ppia*	NM_008907.1	TGGTCTTTGGGAAGGTGAAAG	TGTCCACAGTCGGAAATGGT	109
*Ahcy*	NM_016661.3	AAACCAGGTGATGGCACAGA	CAGCTGGAAGGTGAAGGACA	241
*Bhmt*	NM_016668.3	GAATTCCCCTTTGGATTGGA	CTGATCCAGGGTTTGGTGTG	236
*Cbs*	NM_144855.3	CCCATCCTTGCTGAGTTTGT	TCCAGACTCGATCCTTGTCC	225
*Cidec*	NM_178373.3	AAGATGGCACAATCGTGGAG	TTAGTTGGCTTCTGGGAAAGG	151
*Gnmt*	NM_010321.1	CAGAGTACAAGGCGTGGTTG	CTTTGTCCAGCGTCAACCAG	243
*Hnf4a*	NM_008261.2	ACATTCGGGCAAAGAAGATTG	ACCTGGTCATCCAGAAGGAGTT	127
*Mat1a*	NM_133653.3	TCTGCCAAAGATCTGCCTGT	TATCTGGATGCCCCTCTCCT	207
*Mat2a*	NM_001363798.1	TCCGAGTCTGTAGGGGAAGG	TCAATGGCAGCTCTGGATGT	170
*Nnmt*	NM_001311062.1	CTCTGGCCCCACCATCTATC	CGCCTCAACTTCTCCTCCTT	210
*Pemt*	NM_001290011.1	TAGCGAGATGGGAGCAGAGA	CTGGACAGCACAAACACGAA	226
*Ppargc1a*	NM_008904.2	TTCCCGATCACCATATTCCA	TTCATCCCTCTTGAGCCTTTC	214
*Ppara*	NM_011144	GGGAAAGACCAGCAACAACC	GCAGTGGAAGAATCGGACCT	136
*Pparg*	NM_001127330.1	AGACCACTCGCATTCCTTTG	CCTGTTGTAGAGCTGGGTCTTT	214

### Statistical analysis

Statistical analyses were performed using GraphPad Prism 11 software (GraphPad, La Jolla, CA). Mouse study data were analyzed using a two-way analysis of variance (ANOVA), followed by a Tukey’s post hoc test to assess the effect of fasting or refeeding with *Pparg*^ΔHep^, or the effect of HFD or RSG treatment in the HFD-fed mice with *Pparg*^ΔHep^. An unpaired Student’s t-test was used to compare the effect of RSG in MPH. Data are presented as mean ± standard error of the mean (SEM), and p-values <0.05 were considered significant for all statistical tests.

## Results

### Effect of fasting and refeeding in the regulation of genes involved in the hepatic methionine cycle

**Fig 1:**
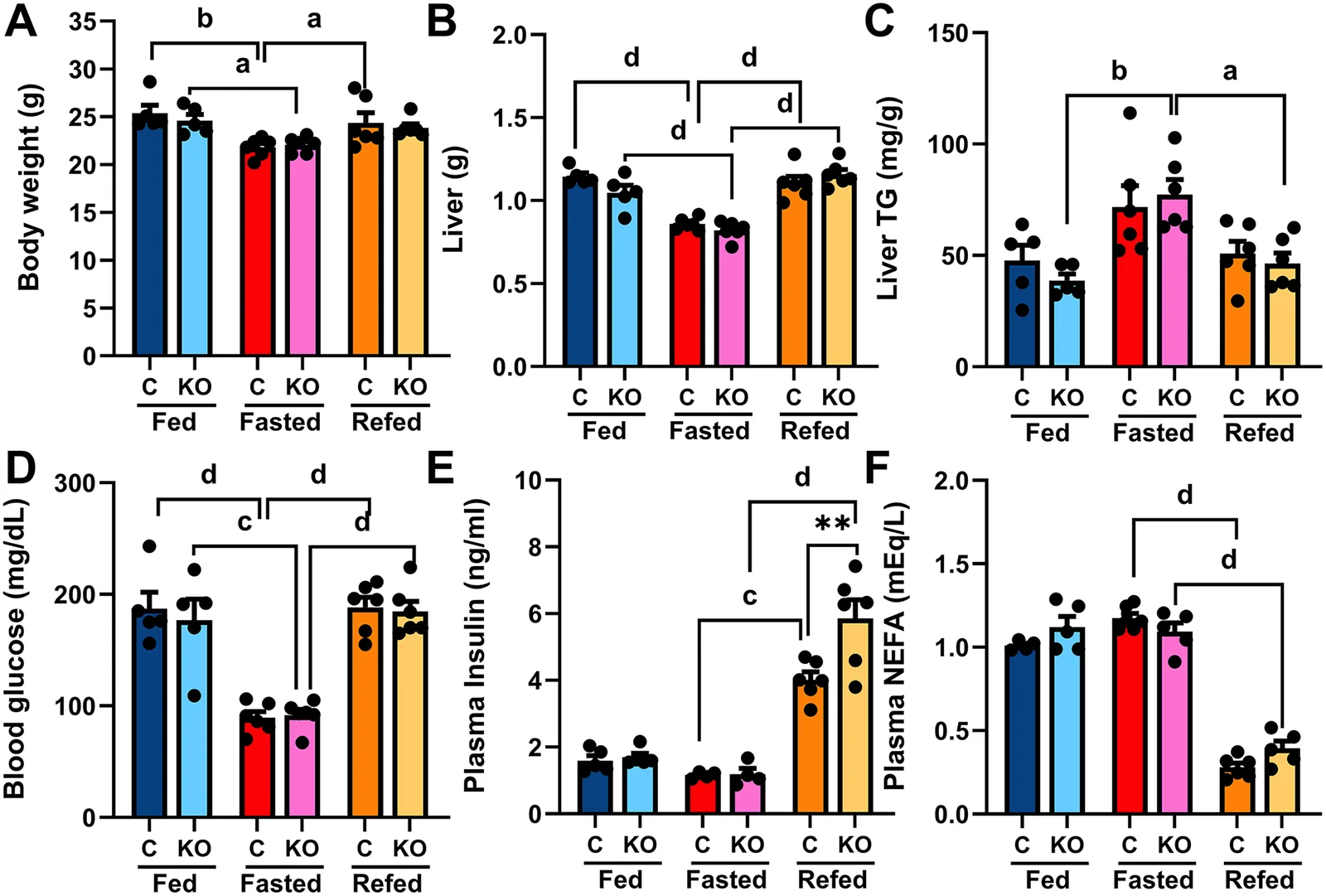
Metabolic effects of fasting and refeeding in control and *Pparg*^ΔHep^ mice. A) body weight, B) liver mass, C) liver triglicerydes (TG) levels, D) blood glucose levels, E) plasma insulin, and F) non-esterified fatty acids (NEFA) levels of male chow-fed control (C) and *Pparg*^ΔHep^ (KO) mice that were euthanized in fed-like conditions (food withdrawn at 0700h), or after 24h fasting (Fasted, food removed at 1200h), or after 6h refeeding that followed a 24h fasting (Refed, food removed at 0700h the previous day, and added 6h before euthanasia). Data are represented as means ± standard error of the mean. Statistical differences (p < 0.05) induced by fasting or refeeding within genotype are indicated by different letters (a, b, c, d). Asterisk indicates statistical differences (p < 0.05) between control and *Pparg*^ΔHep^ mice within a feeding state. n = 4-6 mice/group. a p < 0.05; **, b, p < 0.01; c, p < 0.001; d, p < 0.0001. Data from fed mice in Fig 1A–1F were previously reported by our group [13].

**Fig 2:**
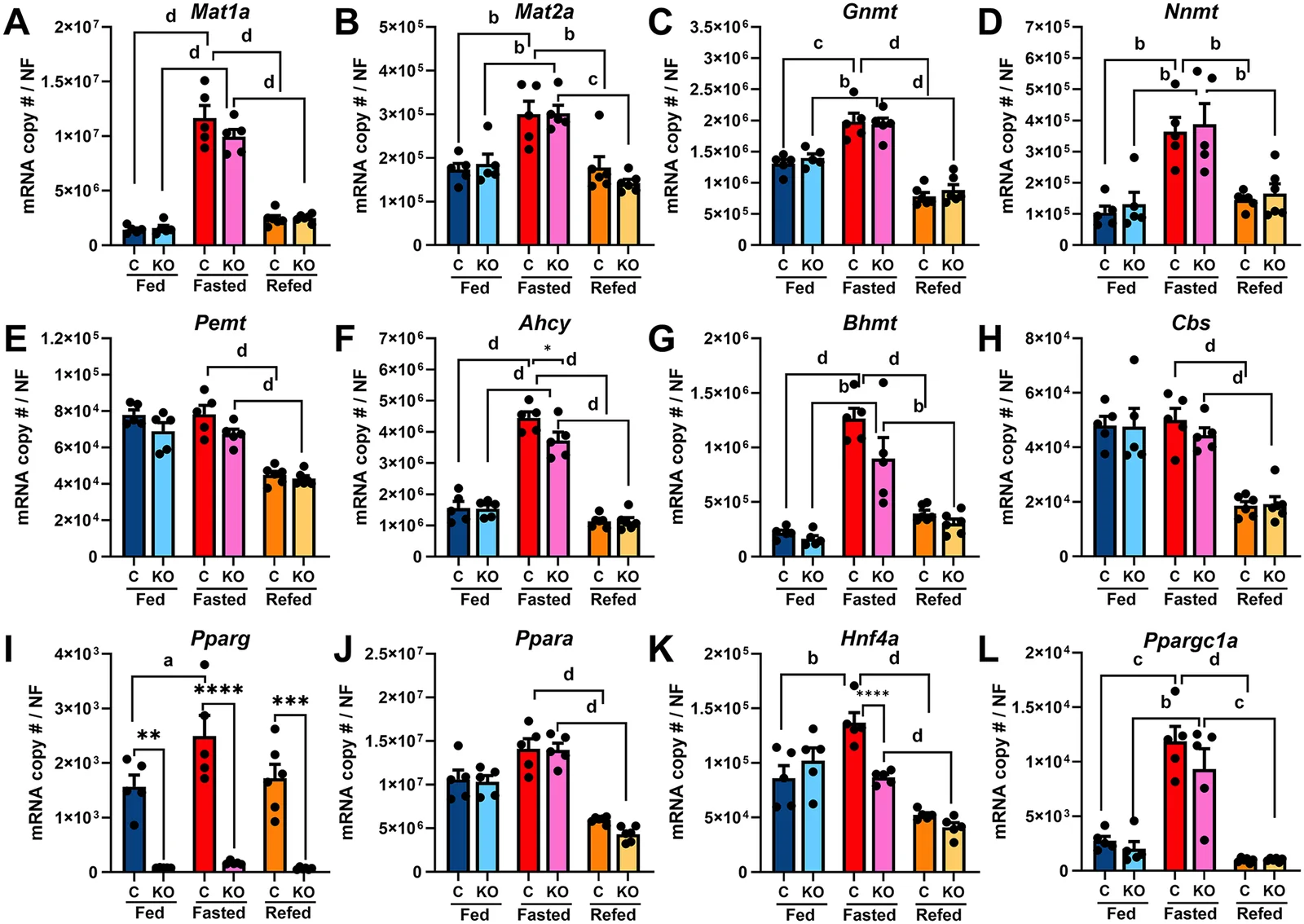
Effects of fasting and refeeding on the hepatic expression of control and *Pparg*^ΔHep^ mice. Expression of A) *Mat1a*, B) *Mat2a*, C) *Gnmt*, D) *Nnmt*, E) *Pemt*, F) *Ahcy*, G) *Bhmt*, H) *Cbs*, I) *Pparg*, J) *Ppara*, K) *Hnf4a*, and L) *Ppargc1a* in male chow-fed control (C) and *Pparg*^ΔHep^ (KO) mice that were euthanized in fed-like conditions (food withdrawn at 0700h), or after 24h fasting (Fasted, food removed at 1200h), or after 6h refeeding that followed a 24h fasting (Refed, food removed at 0700h the previous day, and added 6h before euthanasia). Data are represented as the average of mRNA copy number per sample normalized with a normalization factor (NF) ± standard error of the mean. Statistical differences (p < 0.05) induced by fasting or refeeding within genotype are indicated by different letters (a, b, c, d). Asterisks indicate statistical differences (p < 0.05) between control and *Pparg*^ΔHep^ mice within a feeding state. n = 4-6 mice/group. *, a p < 0.05; **, b, p < 0.01; ***, c p < 0.001; ****, d p < 0.0001. Data of fed mice in Fig 2I was previously reported by our group [13].

Since fasting increases hepatic methionine metabolism, we assessed the effect of *Pparg*^ΔHep^ in fasted and refed mice and compared the metabolic endpoints and gene expression levels to those of their fed-like littermates. The data of the fed control and *Pparg*^ΔHep^ mice in Figs 1 and 2I were previously published [13]. However, the data for these fed mice in Fig 1 are just used as basal (“fed-like”) endpoints to assess the effects of fasting and refeeding in this study. Twenty-four-hour fasting had an overall effect in control and *Pparg*^ΔHep^ mice, decreasing body weight, liver weight, glucose and insulin levels, but increasing liver TG levels (Fig 1A–1F, p-values of two-way ANOVA are in S1 Table). The post hoc analysis of the two-way ANOVA showed that fasting did not significantly increase liver TG in fasted control mice, nor reduce plasma insulin in fasted control and *Pparg*^ΔHep^ mice (Fig 1C–1E). The lack of statistical difference in these endpoints might be due to the duration of fasting in young mice or to comparing fasted and “fed-like” mice after a 4h food withdrawal, rather than to comparing fed mice in the postprandial state. Of note, six hours of refeeding with a chow diet had an overall effect in control and *Pparg*^ΔHep^ mice increasing body weight, liver weight, glucose, and insulin levels, and reducing liver TG levels and plasma NEFA (Fig 1A–1F and S1 Table). These effects on liver and plasma endpoints indicate that nutrients were assimilated and that the mice transitioned from fasting to postprandial conditions. Of note, *Pparg*^ΔHep^ increased the levels of plasma insulin in refed mice (Fig 1E) without altering body weight or liver weight, which could be associated with some degree of insulin resistance due to the loss of hepatocyte *Pparg* expression. Moreover, the two-way ANOVA showed a significant interaction between *Pparg*^ΔHep^ and refeeding on insulin and NEFA levels (Fig 1E and 1F and S1 Table).

In this study, 24h fasting had a significant overall effect on the hepatic expression of *Mat1a*, *Mat2a*, *Gnmt*, *Nnmt*, *Ahcy*, and *Bhmt*, and 6h of refeeding reversed the fasting-induced changes in the expression of these genes (Fig 2A–2G and S1 Table). Moreover, refeeding reduced the expression of *Pemt* and *Cbs*, which was not altered by fasting (Fig 2E and 2H and S1 Table). Of note, peroxisome proliferator-activated receptor alpha (*Ppara*), hepatocyte nuclear factor 4 alpha (*Hnf4a*), and peroxisome proliferator-activated receptor gamma cofactor 1 (*Ppargc1a*) are direct transcriptional activators of genes involved in the hepatic methionine cycle [29,33]. Our two-way ANOVA showed that fasting has an overall effect increasing the expression of *Ppara* and *Ppargc1a*, whereas the post hoc analysis of the two-way ANOVA showed that fasting increases the expression of *Hnf4a* and *Pparg* in control mice, and *Ppargc1a* in control and *Pparg*^ΔHep^ mice (Fig 2I–2L). Here, it should be noted that the data of *Pparg* expression in fed mice in Fig 2I were previously published [13]. By contrast, six hours of refeeding decreased the expression of *Ppara, Hnf4a,* and *Ppargc1a* (Fig 2J–2L and S1 Table), and that downregulation was associated with the reduced expression of genes involved in the methionine cycle in the liver (Fig 2A–2H). Since most of the hepatic expression of *Pparg* is derived from hepatocytes [13]. Given that the expression of hepatic *Pparg* is dramatically reduced in *Pparg*^ΔHep^ mice compared with that of control mice (Fig 2I), we assessed whether the loss of hepatocyte *Pparg* has a positive effect on the expression of genes of the methionine cycle. Interestingly, *Pparg*^ΔHep^ had a significant overall effect on the expression of *Pemt* in fasted and refed mice, and also on the expression of *Ahcy, Bhmt,* and *Hnf4a* in refed mice (Fig 2F, 2G and 2K and S1 Table). The two-way ANOVA showed a significant interaction between *Pparg*^ΔHep^ and refeeding on the expression of *Ahcy* and *Hnf4a* (S1 Table), and that was due to the significant reduction of *Ahcy* and *Hnf4a* by *Pparg*^ΔHep^ in fasted mice. Although *Pparg*^ΔHep^ had a significant effect on the expression of these genes, it did not increase the expression of any hepatic genes involved in the methionine cycle in fasted and/or refed mice. Overall, these results suggest that under fasting and refeeding conditions, the expression of hepatocyte *Pparg* does not contribute to controlling or reducing the hepatic methionine cycle.

### Effect of diet-induced obesity and PPARγ activation in the regulation of genes involved in the hepatic methionine cycle

**Fig 3:**
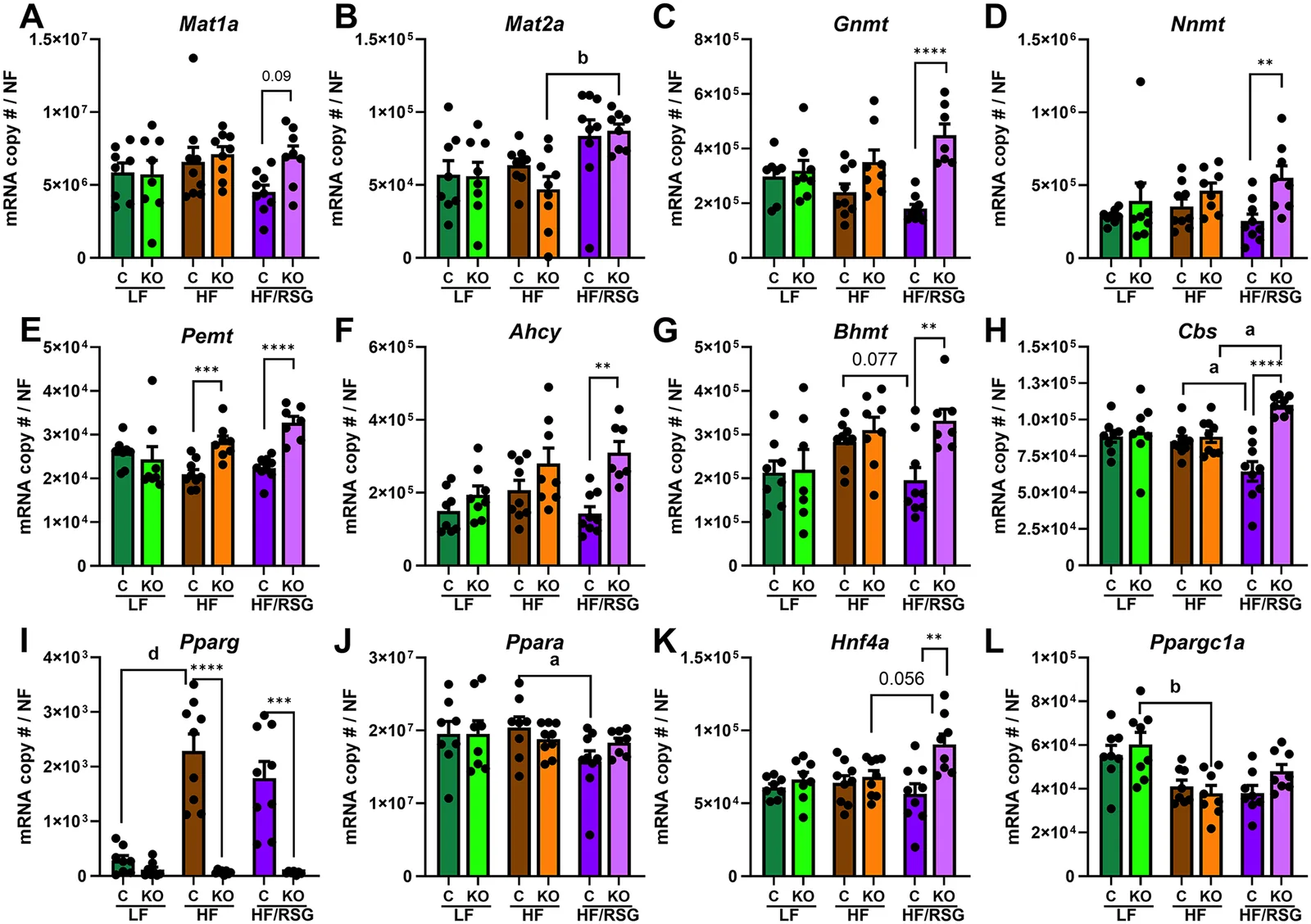
Effects of high-fat diet-induced obesity, and RSG-mediated activation of PPARγ in obese mice on the hepatic expression of control and *Pparg*^ΔHep^ mice. Expression of A) *Mat1a*, B) *Mat2a*, C) *Gnmt*, D) *Nnmt*, E) *Pemt*, F) *Ahcy*, G) *Bhmt*, H) *Cbs*, I) *Pparg*, J) *Ppara*, K) *Hnf4a*, and L) *Ppargc1a* in male control and *Pparg*^ΔHep^ mice that were fed a LF or HF diet for 23 weeks. A subset of HF-fed control (C) and *Pparg*^ΔHep^ (KO) mice were treated with RSG for the last 6 weeks of diet (HF/RSG). Data are represented as the average of mRNA copy number per sample normalized with a normalization factor (NF) ± standard error of the mean. Statistical differences (p < 0.05) induced by HF or RSG within genotype are indicated by different letters (a, b, d). Asterisks indicate statistical differences (p < 0.05) between control and *Pparg*^ΔHep^ mice within a feeding state. n = 7-9 mice/group. a, p < 0.05; **, b p < 0.01; ***, p < 0.001. ****, d p < 0.0001. Data of fed mice in Fig 3I was previously reported by our group [7].

We previously reported that hepatocyte *Pparg* expression is negatively associated with the regulation of methionine metabolism in advanced MASLD/MASH [24]. Here, we have assessed the PPARγ-dependent effects of diet-induced obesity and RSG-mediated PPARγ activation in control and *Pparg*^ΔHep^ mice, which were previously reported by our group [7]. In our previous studies, we showed that HFD increased body weight, liver weight, liver TG, and plasma insulin levels in control mice, and RSG increased body weight and liver steatosis while lowering insulin levels due to its insulin-sensitizing effects [7]. Regarding the effects of diet-induced obesity and RSG on the control of gene expression associated with the hepatic methionine cycle, HFD had an overall effect increasing only the expression of hepatic *Ahcy* and *Bhmt* (Fig 3F and 3G and S1 Table), and RSG had an overall effect increasing *Mat2a* and *Pemt* (Fig 3B and 3E and S1 Table). Moreover, *Pparg*^ΔHep^ had an overall effect increasing the expression of *Mat1a, Gnmt*, *Nnmt*, *Pemt*, *Ahcy*, *Bhmt*, and *Cbs* in RSG-treated mice (Fig 3A and 3C–3H and S1 Table). Of note, the two-way ANOVA showed a significant interaction between *Pparg*^ΔHep^ and RSG-treatment in the expression of *Gnmt*, *Bhmt*, and *Cbs* (Fig 3C, 3G and 3H and S1 Table). The post hoc analysis of the two-way ANOVA showed that RSG increased the expression of *Mat2a* and *Cbs* in *Pparg*^ΔHep^ mice, and reduced that of *Bhmt* (p = 0.077) and *Cbs* in control mice (Fig 3B,3G and 3H). Moreover, in the HF-fed mice, *Pparg*^Δhep^ increased the expression of *Pemt*, and in RSG-treated mice *Pparg*^Δhep^ increased the expression of *Mat1a* [p = 0.09], *Gnmt, Nnmt, Pemt, Ahcy, Bhmt, and Cbs* (Fig 3A–3H). These data might indicate that RSG-mediated activation of PPARγ in hepatocytes could negatively regulate the expression of genes involved in the methionine cycle in the liver of diet-induced obese control (*Pparg*-intact) mice but not in *Pparg*^Δhep^ mice. The expression of hepatic *Pparg* was significantly increased by HFD in control mice (Fig 3I, these data were previously published [7]), and in this analysis, we identified a negative overall effect of HFD on the expression of *Ppargc1a*, and also of RSG on the expression of *Ppara* (Fig 3J and 3L). The post hoc analysis of the two-way ANOVA showed that HFD reduced the expression of *Ppargc1a* in *Pparg*^ΔHep^ mice, and RSG reduced the expression of *Ppara* in control mice, and increased the expression of hepatic *Hnf4a* in *Pparg*^Δhep^ mice (Fig 3J–3L). In addition, *Pparg*^ΔHep^ increased the expression of *Hnf4a* in RSG-treated mice (Fig 3K), and this could be associated with a positive regulation of most of the genes involved in the hepatic methionine cycle (Fig 3A–3H). Overall, the positive effects of RSG in the expression of *Mat1a, Gnmt*, *Nnmt*, *Pemt*, *Ahcy*, *Bhmt*, and *Cbs,* and that of *Hnf4a,* may be indicative of a negative association between hepatocyte *Pparg* and the regulation of the hepatic methionine cycle. As we previously reported [7], RSG-treated diet-induced obese *Pparg*^ΔHep^ mice show a significant improvement in liver health, which may be associated with enhanced effects of RSG-mediated insulin sensitization in the regulation of hepatic methionine metabolism.

**Fig 4:**
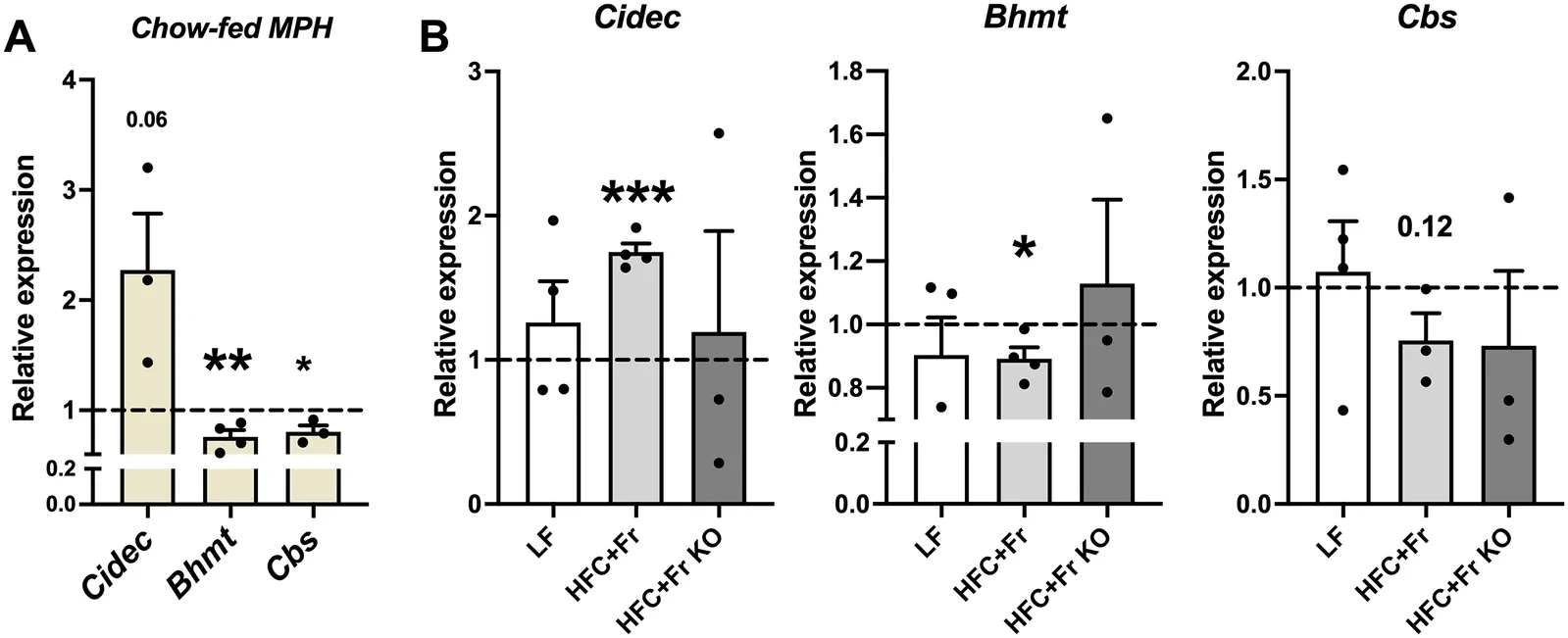
Effects of RSG-mediated activation of hepatocyte PPARγ on the gene expression of mouse primary hepatocytes. Expression of *Cidec*, *Bhmt*, and *Cbs* in mouse primary hepatocytes of chow-fed Pparg^fl/fl^ mice (A), LF-fed control, HFC + Fr-fed control, and HFC + Fr-fed *Pparg*^ΔHep^ (KO) mice (B). Data are represented as the average of relative gene expression values ± standard error of the mean against their vehicle (Veh)-treated mouse primary hepatocytes (set as a dashed line line at 1). Asterisks indicate statistical differences between RSG-treated MPH vs vehicle-treated MPH. *, p < 0.05. **, p < 0.01. ***, p < 0.001. n = 3-4/group.

To further investigate the role of hepatocyte PPARγ in the regulation of genes involved in the hepatic methionine cycle under diet-induced metabolic stress, we isolated MPH from metabolically healthy 4.5-month-old chow-fed and 9- month-old LFD-fed mice, and from obese, metabolically unhealthy 9-month-old HFC + Fr-fed control and *Pparg*^ΔHep^ mice. In these MPH, we assessed the effect of RSG-mediated activation of hepatocyte PPARγ in the regulation of some hepatocyte genes. Notably, RSG increased the expression of the PPARγ-target gene cell death-inducing DFFA-like effector c (*Cidec*) in MPH of chow-fed mice (Fig 4A) and HFC + Fr-fed control mice (Fig 4B), similar to what we published previously with a higher dose of RSG [24]. Interestingly, RSG reduced the expression of *Bhmt* in MPH from chow-fed mice, and HFC + Fr-fed control mice (Fig 4A and 4B). Furthermore, RSG reduced the expression of *Cbs* in chow-fed mice and showed a trend toward reducing it in HFC + Fr-fed control mice (Fig 4A and 4B). The RSG-mediated regulation of *Cidec*, *Bhmt*, and *Cbs* was not observed in MPH obtained from HFC + Fr-fed *Pparg*^ΔHep^ mice. These data suggest that RSG-mediated activation of PPARγ in hepatocytes may directly reduce the expression of *Bhmt* and *Cbs*, thereby altering hepatocyte methionine cycle activity.

## Discussion

Most SAM-dependent methylation reactions take place in the liver and play a key role in maintaining liver health [1,6,34,35]. The enzymes that generate SAM, as well as many SAM-dependent methyltransferases, are highly expressed in hepatocytes [34–36]. In hepatocytes, cellular stress and fatty acids activate key transcription factors and nuclear receptors, regulating the expression of genes involved in the methionine cycle and maintaining cell health. Fasting, refeeding, and diet-induced steatosis can induce cellular stress in hepatocytes and alter the flux of fatty acids to the liver, thereby activating key transcription factors involved in regulating the methionine cycle. In this study, we have reproduced and extended the knowledge of the expression of genes involved in methionine cycle in the liver under fasting, refeeding, and diet-induced steatosis, and show that expression of hepatocyte *Pparg* does not reduce the expression of genes involved in the hepatic methionine cycle in the context of fasting and refeeding, but it could reduce the expression of key methyltransferases in conditions of obesity and insulin resistance.

It is known that fasting enhances hepatic methionine metabolism by increasing the expression of *Mat1a* and major methyltransferases [27–29]. Our study shows that both *Mat1a* and *Mat2a* expression is increased in fasted livers, although previous reports reported that *Mat2a* was not increased by fasting [28,29]. Of note, Capelo-Diz *et al.* reported that increased methylation reactions during fasting may be required to prevent liver damage due to increased metabolism in fasting conditions and to maintain mitochondrial oxidative capacity and ATP production [27]. Here, we report that fasting increases the expression of major SAM-dependent methyltransferases, including *Gnmt* and *Nnmt*, which may consume the SAM produced by MAT1A activity in the hepatocytes during fasting. Also, we observed that fasting increases the expression of hepatic *Ahcy* and *Bhmt*, which will convert SAH to homocysteine and back to methionine [29,37]. Increased activity of SAM-dependent methyltransferases may raise homocysteine levels, which can induce endoplasmic reticulum stress and dysregulate lipid metabolism [38], so the upregulation of *Bhmt* will maintain liver health. Interestingly, our study shows that fasting does not increase the expression of hepatic *Pemt* and *Cbs*, which might indicate that use of SAM for hepatic VLDL or use of homocysteine for glutathione production during fasting does not require increased *Pemt* or *Cbs* expression, or that these enzymes may not significantly deplete SAM and homocysteine pools during fasting, respectively. Overall, we have reproduced the effect of fasting in the control of *Mat1a*, *Ahcy*, *Bhmt*, and *Cbs* as previously reported by others [29,37], and extended it to additional genes (*Mat2a*, *Gnmt*, *Nnmt*, and *Pemt*). In addition, our study shows that refeeding (postprandial phase) reverses the fasting effect on most of these genes and reduces the basal expression of hepatic *Pemt* and *Cbs,* which might impact VLDL secretion rate and glutathione production. The regulation of the genes involved in the hepatic methionine cycle may be produced by activation of *Ppargc1a* and *Hnf4a* during fasting, which are known to activate the expression of some of these genes such as *Mat1a* and *Bhmt* [29,33]. Our study also reveals that in lean mice, despite the fasting-associated influx of fatty acids that might activate hepatocyte PPARγ [39], the expression of *Pparg* in hepatocytes does not attenuate the effects of fasting and refeeding on the expression of genes involved in the methionine cycle.

Diet-induced obesity also regulates hepatic methionine metabolism [8]. High-fat diets increase body weight, plasma insulin levels, and liver steatosis in C57BL/6J male mice [7], and these diets may promote the onset of MASLD. MASLD may progress to MASH, which is strongly associated with the negative regulation of hepatic methionine metabolism by affecting the production of SAM, expression of SAM-dependent methyltransferases, and the remethylation or transulfuration of homocysteine [8,16,24]. Specifically, a western diet with 42% Kcal from fat reduces the expression of hepatic *Mat1a*, *Mat2a*, *Gnmt*, and *Cbs* gene in female C57Bl/6J mice [8], a high fat diet with 45% Kcal from fat increases the expression of hepatic *Bhmt2* and decreases that of *Pemt* and *Cbs* in male C57BL/6J mice [40], and a high fat diet with 60% Kcal from fat increases hepatic *Bhmt* and trended to reduce *Cbs* gene expression in male C57Bl/6N mice [41]. We assessed the expression of these genes in male C57Bl/6J mice fed a high-fat diet with 60% Kcal from fat [7] and observed an overall positive effect of HFD on *Ahcy* and *Bhmt* expression. Furthermore, we observed that RSG-mediated insulin-sensitizing effects and activation of hepatocyte PPARγ reduced the expression of hepatic *Bhmt* and *Cbs*, potentially negatively impacting the remethylation and transsulfuration of homocysteine. The negative regulation of *Bhmt* and *Cbs* was *Pparg*-dependent and could contribute to the increase of homocysteine in hepatocytes, as we have reported [8,16,24], and that might induce cellular stress in the liver [38]. Noteworthy, this *Pparg*-dependent *in vivo* effect was not associated with a downregulation of *Ppargc1a*, *Hnf4a*, or *Ppara* expression that are known to increase *Bhmt* expression. In fact, this effect seems to be direct as MPH treated with RSG showed a decreased expression of *Bhmt* and *Cbs*. Moreover, the RSG-mediated insulin-sensitizing effects in these mice, as reported previously [7], could have a positive effect in *Pparg*^ΔHep^ mice because the expression of *Hnf4a*, and *Cbs* is upregulated above that of HF-fed *Pparg*^ΔHep^ mice. Of note, *Pparg* and *Hnf4a* axis could play an antagonist role controlling hepatocyte gene expression of key methyltransferases in conditions of steatosis, as both nuclear receptors bind *Bhmt* gene [42], and *Hnf4a* can increase *Bhmt* expression [29]. Taken together, this data show that pharmacological activation of hepatocyte PPARγ negatively impacts the regulation of key genes involved in the methionine cycle in hepatocytes, which might reduce the ability of the cell to mediate transmethylation reactions that use SAM, and to reduce the levels of homocysteine that is processed by BHMT and CBS to prevent liver damage.

In conclusion, our study describes the strict regulation of the hepatic methionine cycle by fasting and refeeding that controls the expression of genes involved in the synthesis of SAM, the transmethylation of SAM, and the final remethylation or transsulfuration of homocysteine, where these changes seem to be independent of hepatocyte *Pparg*. Although diet-induced obesity does not impose a strong regulation of the hepatic methionine cycle, the expression of *Pemt*, *Gnmt, Nnmt, Ahcy, Bhmt, Cbs, and Hnf4a* was increased upon RSG-mediated insulin sensitization in diet-induced obese mice without hepatocyte *Pparg* expression. Since the progression of MASLD is associated with increased homocysteine levels [8,16,24,43–45], and homocysteine increases the risk of advanced hepatic fibrosis in patients with alcoholic liver disease [46,47], hepatocyte PPARγ activation may have negative consequences for liver health, and its activity in hepatocytes should be considered when PPAR agonist are used in the clinic.

## Supporting information

S1 Table Summary of p-values of two-way ANOVA in Figs 1–3. (DOCX)
