# Supplementary material for: PPARγ-dependent and -independent regulation of genes involved in hepatic methionine cycle in fasted or diet-induced obese mice
Source: PLoS One. 2026 Sep 18;21(9):e0358698. doi: 10.1371/journal.pone.0358698 (PMC13588356; doi:10.1371/journal.pone.0358698)
Supplement: Supplementary file 1 [file pone.0358698.s001.docx]

**Summary tables of two-way ANOVA results (p-values) of endpoints assessed in Figure 1**

|  | **BW** | **Liver** | **Liv TG** | **Glucose** | **Insulin** | **NEFA** |
| --- | --- | --- | --- | --- | --- | --- |
| **Interaction** | 0.3668 | 0.2838 | 0.3309 | 0.6006 | 0.7886 | 0.0512 |
| ***Pparg*^ΔHep^** | 0.6702 | 0.0228 | 0.8218 | 0.7332 | 0.6288 | 0.7445 |
| **Fasting** | <0.0001 | <0.0001 | 0.0004 | <0.0001 | 0.0035 | 0.1528 |
|  |  |  |  |  |  |  |
|  | **BW** | **Liver** | **Liv TG** | **Glucose** | **Insulin** | **NEFA** |
| **Interaction** | 0.5265 | 0.1288 | 0.4665 | 0.7003 | 0.0323 | 0.0196 |
| ***Pparg*^ΔHep^** | 0.8431 | 0.7989 | 0.937 | 0.921 | 0.0277 | 0.6725 |
| **Refed** | 0.0021 | <0.0001 | 0.0012 | <0.0001 | <0.0001 | <0.0001 |

**Summary tables of two-way ANOVA results (p-values) of the endpoints assessed in Figure 2**

| **Source of variation** | ***Mat1a*** | ***Mat2a*** | ***Gnmt*** | ***Nnmt*** | ***Pemt*** | ***Ahcy*** | ***Bhmt*** | ***Cbs*** | ***Pparg*** | ***Ppara*** | ***Hnf4a*** | ***Ppargc1a*** |
| --- | --- | --- | --- | --- | --- | --- | --- | --- | --- | --- | --- | --- |
| **Interaction** | 0.194 | 0.8106 | 0.4726 | 0.9757 | 0.8047 | 0.1051 | 0.1716 | 0.5745 | 0.0726 | 0.9357 | 0.0031 | 0.4665 |
| ***Pparg*^ΔHep^** | 0.2607 | 0.7369 | 0.7982 | 0.5856 | 0.0205 | 0.0863 | 0.0706 | 0.5169 | <0.0001 | 0.8389 | 0.0924 | 0.1958 |
| **Fasting** | <0.0001 | <0.0001 | <0.0001 | <0.0001 | 0.8567 | <0.0001 | <0.0001 | 0.8945 | 0.0334 | 0.0016 | 0.0799 | <0.0001 |
|  |  |  |  |  |  |  |  |  |  |  |  |  |
| **Source of variation** | ***Mat1a*** | ***Mat2a*** | ***Gnmt*** | ***Nnmt*** | ***Pemt*** | ***Ahcy*** | ***Bhmt*** | ***Cbs*** | ***Pparg*** | ***Ppara*** | ***Hnf4a*** | ***Ppargc1a*** |
| **Interaction** | 0.1722 | 0.3948 | 0.4527 | 0.9661 | 0.1369 | 0.0468 | 0.1865 | 0.2897 | 0.1504 | 0.2637 | 0.0021 | 0.231 |
| ***Pparg*^ΔHep^** | 0.1991 | 0.4505 | 0.7726 | 0.5906 | 0.0375 | 0.0445 | 0.0355 | 0.4057 | <0.0001 | 0.1989 | <0.0001 | 0.2454 |
| **Refed** | <0.0001 | <0.0001 | <0.0001 | <0.0001 | <0.0001 | <0.0001 | <0.0001 | <0.0001 | 0.065 | <0.0001 | <0.0001 | <0.0001 |

**Summary tables of two-way ANOVA results (p-values) of the endpoints assessed in Figure 3**

| **Source of variation** | ***Mat1a*** | ***Mat2a*** | ***Gnmt*** | ***Nnmt*** | ***Pemt*** | ***Ahcy*** | ***Bhmt*** | ***Cbs*** | ***Pparg*** | ***Ppara*** | ***Hnf4a*** | ***Ppargc1a*** |
| --- | --- | --- | --- | --- | --- | --- | --- | --- | --- | --- | --- | --- |
| **Interaction** | 0.6846 | 0.3566 | 0.2364 | 0.9795 | 0.0181 | 0.6369 | 0.7292 | 0.9816 | <0.0001 | 0.5813 | 0.885 | 0.3413 |
| ***Pparg*^ΔHep^** | 0.8076 | 0.2954 | 0.0856 | 0.1425 | 0.1079 | 0.0598 | 0.5743 | 0.5266 | <0.0001 | 0.5859 | 0.2866 | 0.8524 |
| **HFD** | 0.2031 | 0.885 | 0.7411 | 0.3385 | 0.8016 | 0.0229 | 0.0112 | 0.5042 | <0.0001 | 0.966 | 0.5666 | 0.0002 |
|  |  |  |  |  |  |  |  |  |  |  |  |  |
| **Source of variation** | ***Mat1a*** | ***Mat2a*** | ***Gnmt*** | ***Nnmt*** | ***Pemt*** | ***Ahcy*** | ***Bhmt*** | ***Cbs*** | ***Pparg*** | ***Ppara*** | ***Hnf4a*** | ***Ppargc1a*** |
| **Interaction** | 0.1785 | 0.2162 | 0.0249 | 0.1218 | 0.209 | 0.1323 | 0.0437 | <0.0001 | 0.3183 | 0.0765 | 0.0159 | 0.0537 |
| ***Pparg*^ΔHep^** | 0.0413 | 0.4158 | <0.0001 | 0.0017 | <0.0001 | 0.0005 | 0.0036 | <0.0001 | <0.0001 | 0.6691 | 0.0028 | 0.3212 |
| **RSG** | 0.129 | 0.0006 | 0.5813 | 0.9127 | 0.0204 | 0.5732 | 0.2072 | 0.8978 | 0.2978 | 0.033 | 0.2129 | 0.2903 |
